# Modern Strategies for Osteoporosis Therapy: Current Status and Prospects for Targeted Intervention

**DOI:** 10.3390/ijms262211092

**Published:** 2025-11-16

**Authors:** Vitalii Omelchenko, Vladimir Koval, Natalya Slazhneva, Natalya Bondarenko, Elizaveta Shatunova, Mariya Vorobyeva, Maxim Korolev

**Affiliations:** 1 Institute of Cytology and Genetics, Siberian Branch of Russian Academy of Sciences, Novosibirsk 630090, Russia; sitnikovanat9@gmail.com (N.S.); bond802888@yandex.ru (N.B.); lizashatunova@yandex.ru (E.S.); 2 Research Institute of Clinical and Experimental Lymphology—Branch of the Institute of Cytology and Genetics, Siberian Branch of Russian Academy of Sciences, Novosibirsk 630117, Russia; koval.v.v97@gmail.com (V.K.); kormax@bk.ru (M.K.); 3 Institute of Chemical Biology and Fundamental Medicine, Siberian Branch of Russian Academy of Sciences, Novosibirsk 630090, Russia; mvorobjeva@1bio.ru

**Keywords:** osteoporosis, pathogenesis, WNT pathway, therapy, targeted inhibition, antiosteoporotic drugs, antibodies, siRNAs, aptamers, small molecules

## Abstract

Osteoporosis is becoming a “silent pandemic” because of its ever-increasing prevalence and the absence of clinical manifestations until a bone fracture happens. The purpose of this review is to summarize the actual data on the pathogenesis of osteoporosis and its treatment options. The disease develops through a multifactorial process involving an imbalance between bone remodeling and different factors like genetics, non-coding RNA regulation, osteoimmune dysregulation, oxidative stress, cellular senescence, and fat–bone interactions. Existing medications have beneficial effects by preserving and increasing bone density and reducing the risk of fractures. Among them, there are bisphosphonates, strontium ranelate, calcitonin, estrogen-progestin therapy, selective estrogen receptor modulators, and parathyroid hormone analogues. Otherwise, they suffer from certain disadvantages, such as adverse effects, including serious ones, and limitations associated with comorbidity. Targeting pathways underlying bone metabolism could significantly improve the therapeutic options and provide new tools in the fight against osteoporosis. We consider here targeted therapeutics that are already in clinical practice, as well as the most promising novel agents that are now under development: antibodies, siRNAs, aptamers, and small molecules.

## 1. Introduction

Osteoporosis (OP) is a metabolic disease of the skeleton characterized by a high risk of fractures with minimal trauma due to a decrease in bone mass, microarchitectural disruption, bone mineralization, and bone strength [1]. The social significance of osteoporosis is determined by the high risk of fractures of the vertebral bodies and long skeleton bones with minimal trauma, which leads to high material costs for treatment, high levels of incapacity for work, disability, and mortality. The insidious nature of the disease lies in the absence of symptoms until a fracture occurs, which makes it difficult to argue for prevention and diagnosis of the disease.

It is quite difficult to estimate the real global OP prevalence due to the lack of clinical manifestations before fracture formation or before imaging. The prevalence of OP and osteopenia worldwide is 19.7% and 40.4%, respectively, and varies greatly depending on the country (from 4.1% in the Netherlands to 52.0% in Turkey) and continent (from 8.0% in Oceania to 26.9% in Africa) [2]. Among women, the global OP prevalence is 23.1%, while among men, this figure reaches only 11.7% [3]. The annual number of fractures associated with bone fragility is growing and now exceeds 75 million cases per year [4,5].

The treatment costs currently make osteoporosis one of the major public health problems. The first year (especially in the first 6 months) after a fracture has a high mortality rate associated with immobilization, thromboembolic, and infectious complications. Patients experience a significant decrease in their life quality; many of them prominently reduce or lose their self-care ability. Falls and related fractures place a heavy economic burden on the healthcare system [6]. The cost of treating fractures accounts for 66% of the total amount, pharmacological prevention for 5%, and long-term fracture treatment for 29% [7]. In 2015, direct medical costs totaled $637.5 million for fatal fall injuries and $31.3 billion for non-fatal ones. That same year, hospitalizations averaged $30,550 per fall-related hospitalization, totaling $17.8 billion, and costs are projected to increase [8].

OP therapy mainly aims to prevent fractures by increasing bone mass and bone strength [9]. Existing drugs address different components of the pathogenesis pathway. Some drugs predominantly show antiresorptive properties (e.g., bisphosphonates, denosumab, calcitonin, and menopausal hormone therapy) by acting on osteoclasts, while others produce an anabolic effect (parathyroid hormone analogues and romosozumab) by enhancing bone formation [10]. In addition to their proven therapeutic effect, these medications also have disadvantages that limit their use [11]. Although bisphosphonates remain the first-line therapy for osteoporosis, their long-term use may be associated with fractures due to excessive bone fragility, and their use is contraindicated in patients with low renal function [12]. The use of strontium ranelate as a routine OP treatment for osteoporosis is discouraged due to severe skin reactions such as toxic epidermal necrolysis, cardiovascular disease (myocardial infarction), and thromboembolic complications. There is a possible association between calcitonin treatment and malignant processes [13]. Estrogen replacement therapy/hormone replacement therapy (ERT/HRT) fails to qualify as first-line therapy because their potential adverse events (high risk of cardiovascular disease, stroke, venous thromboembolism, and invasive breast cancer) and the health risks exceed the benefits. Selective estrogen receptor modulators (SERMs) exhibit an analogous spectrum of adverse effects. Well-tolerated Denosumab therapy is limited by the high treatment cost as well as the high risk of fractures upon discontinuation of treatment [5]. The use of parathyroid hormone analogues for more than two years is not recommended due to a dose-dependent increase in the osteosarcoma formation risk in animal models [14]. Romosozumab has been shown to have a high cardiovascular risk, so its long-term safety is unclear [15]. These considerations underscore the need to search for new therapeutic options for osteoporosis management, with particular emphasis on targeted treatment strategies. Several classes of molecules (antibodies, siRNAs, aptamers, and small molecules) have significant potential in OP therapy by targeting the Wnt pathway through various mechanisms. Therefore, the development of new, highly effective and safe pharmacological methods for OP treatment and prevention remains an essential scientific and medical task aimed at reducing mortality, preventing disability, and prolonging active longevity. We consider here targeted therapeutics that are already in clinical practice, as well as the most promising novel agents that are now under development. In this review, we focus more on OP treatment than on prevention strategies, and consider osteoporosis as a primary disease, without addressing alterations in bone mineral density that occur as complications of immune-mediated inflammatory rheumatic disorders.

## 2. Clinical Aspects of Osteoporosis

**Figure 1:**
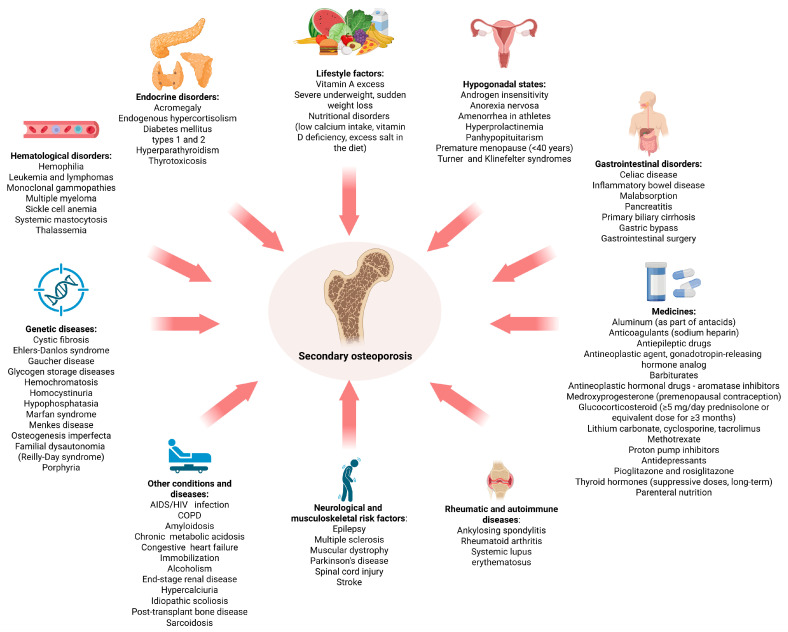
Conditions and diseases leading to secondary osteoporosis.

Osteoporosis is classified as primary and secondary [10,16]. Primary OP represents an independent disease that predominates in postmenopausal women with decreased estrogen secretion and in men over the age of 50, in whom sex hormone-binding globulin inactivates testosterone and estrogen as they age, which can reduce bone mineral density (BMD) over time [1]. There are also distinct, extremely rare forms of primary OP, such as idiopathic osteoporosis in premenopausal women or men under 50 and juvenile osteoporosis in children. Secondary OP is caused by a variety of conditions, diseases, and medications, as summarized in Figure 1.

OP has no clinical signs until a fracture occurs. In terms of prognosis, the most important fractures are femoral neck and vertebral ones. Other locations are also possible; one of the most common is a radius fracture. All fractures can occur with minimal trauma (falling from a standing height) and habitual everyday activities (e.g., opening a window, coughing, etc.) [5,17].

The gold standard for diagnosing osteoporosis is dual-energy X-ray absorptiometry (DXA), which determines bone density. Three areas are routinely diagnosed: the lumbar spine and both femurs. When it is impossible to examine a particular area (prosthetic joint, metal implants in the spine, etc.), one-third (33%) of the radius is additionally assessed. This location, however, is not very indicative for verifying systemic BMD reduction and assessing fracture risks, since bone density can decrease locally in this area in various diseases (e.g., inflammatory rheumatic ones). DXA has several limitations. For example, osteoarthritis of the spine or hip joint is common in older adults and may overestimate BMD values without reflecting skeletal strength. Alternatively, the osteoproliferation associated with ankylosing spondylitis inflates the DXA values, masking the increased risk of fractures. Bone tissue heterogeneity and its quality deterioration can be determined using the Trabecular Bone Score (TBS) and can be excluded from the scan by the operator [18].

The results are compared with the corresponding population reference standards and presented as a quantitative value of BMD (g/cm^2^) and T-score/Z-criterion (the T-criterion is used for postmenopausal women, perimenopausal women, and men over 50, and the Z-criterion is used for younger people) [18]. Diagnostic classification uses the lowest T-score in any of the recommended areas for DXA. According to the World Health Organization (WHO) criteria [19], the diagnosis of osteoporosis is verified when the T-score is −2.5 SD or less. In addition, osteoporosis can be diagnosed and a treatment strategy developed if the patient has a history of low-energy fractures. A T-score between −2.5 and −1.0 is defined as “osteopenia”, “low bone mass”, or “low bone density”. In children and adolescents, premenopausal women, and men under the age of 50, the use of the Z-score is recommended [20]. A Z-score ≤ −2.0 is defined as “bone mineral density below the expected range for a given age”. It is assumed that in such patients, the terms “osteopenia” or “osteoporosis” should not be used to classify bone mineral density indicators [18]. Instrumental measurement of bone density is critical for diagnosis and treatment monitoring, since BMD decrease exponentially increases the fracture risk, and there are no clinical precursors of low bone mass. In turn, an increase in BMD can serve as a surrogate marker of treatment efficacy, as a strong correlation has been demonstrated between an increase in BMD after treatment and a reduction in the fracture risk [21]. The minimum goal is to increase the T-score by more than −2.5 SD, but in the presence of a history of fractures, this goal can be tightened to −2.0 SD and even −1.5 SD [9,22].

If DXA is not available, the 10-year fracture probability can be assessed with the FRAX (Fracture Risk Assessment Tool) questionnaire, which is developed for each specific region [23]. The assessment uses the factors that significantly increase the fracture risk: age, gender, low body mass index, previous fractures, hip fractures in parents, treatment with glucocorticoids, current smoking, alcohol consumption of 3 or more units per day, and causes of secondary osteoporosis [5]. FRAX determines the overall risk of osteoporotic fracture (including spine, shoulder, forearm, and hip) and the risk of hip fracture. High gives the grounds for starting treatment [16,23]. We may also suspect osteoporosis in cases of significant height loss (≥2 cm over 1–3 years or ≥4 cm over a lifetime) and thoracic kyphosis [5]. Moreover, a number of societies propose verifying osteoporosis in the presence of a previous low-trauma major osteoporotic fracture, even with normal BMD (hip, spine, forearm, humerus, pelvis) [24].

Bone mass can also be assessed by quantitative computed tomography and ultrasound, but they are less common due to various limitations.

Standard biochemical markers of bone formation include total alkaline phosphatase (ALP), bone-specific alkaline phosphatase (BALP), osteocalcin (OC), procollagen type 1 N-terminal propeptide (P1NP), and procollagen type 1 C-terminal propeptide (P1CP), and for bone resorption—hydroxyproline (HYP), hydroxylysine (HYL), deoxypyridinoline (DPD), pyridinoline (PYD), bone sialoprotein (BSP), osteopontin (OP), tartrate-resistant acid phosphatase 5b (TRAP 5b), carboxy-terminal crosslinked telopeptide of type 1 collagen (CTX-1), amino-terminal crosslinked telopeptide of type 1 collagen (NTX-1), and cathepsin K (CTSK). We should also mention the regulators of bone metabolism, namely receptor activator of NF-kB ligand (RANKL), osteoprotegerin (OPG), Dickkopf-1 (DKK-1), and sclerostin. They can serve as surrogate markers for fracture risk in situations where BMD cannot be assessed or when attempting to assess bone metabolism dynamics in the short-term [5,25].

## 3. Key Mechanisms and Regulatory Pathways in Osteoporosis Pathogenesis

**Figure 2:**
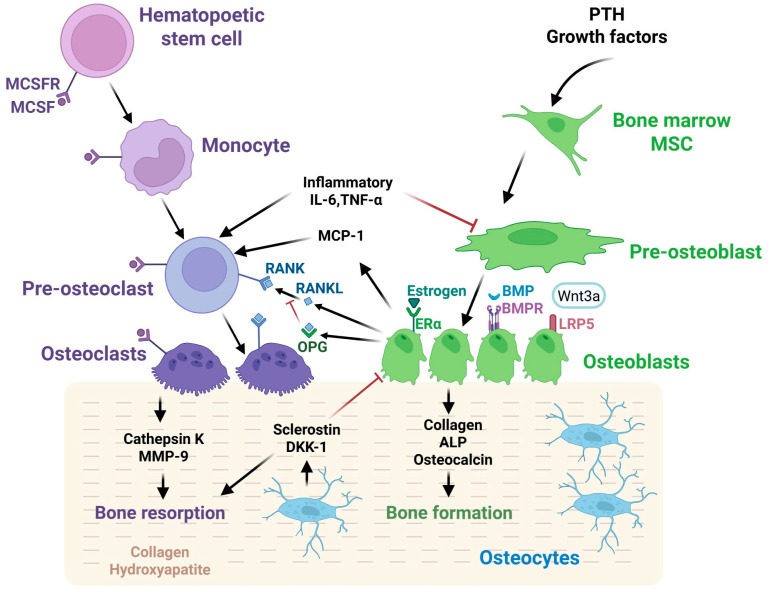
Key pathways and players in bone resorption and synthesis balance.

The state of the skeleton and the quality of bone tissue are directly related to the functional activity of osteoblasts, osteoclasts, and osteocytes, as well as to the bone matrix containing collagen proteins, glycosaminoglycans, and glycoproteins [26]. During the continuous process of bone remodeling, osteoclasts provide bone resorption, while osteoblasts are responsible for its formation (Figure 2).

Normally, bone resorption and synthesis processes balance one another. Bone remodeling ensures the physiological, continuous renewal of the bone matrix. It begins with an activation phase, when parathyroid hormone (PTH) binds to its receptor on preosteoblasts through endocrine signals [27]. At the next phase of bone resorption, PTH causes osteoblasts to release monocyte chemotactic protein 1 (MCP-1), which induces preosteoclast migration. At the same time, osteoblasts increase the production of macrophage colony stimulating factor (MCSF-1) and receptor activator of NF-κB ligand (RANKL) and decrease the production of osteoprotegerin (OPG), thus promoting the differentiation of pre-osteoclasts into osteoclasts. Osteoclasts then form areas of degradation of the mineralized bone matrix. During the stage of reversion, cells remove demineralized collagen from the bone surface. This stops the resorption process and initiates the formation of bone tissue. Osteocytes form deposited osteoid, which subsequently mineralizes, thus finalizing the remodeling cycle [27].

Conversely, osteoporosis is characterized by an imbalance in bone remodeling, with the balance shifting in favor of resorption. As a result, osteoporosis causes bone to break down faster and in greater quantities, while the reduced activity of osteoblasts makes new bone form more slowly or in insufficient quantities.

Bone tissue homeostasis is governed by the regulatory pathways of osteoblastogenesis (canonical WNT signaling pathway, bone morphogenetic protein (BMP) signaling pathway) and osteoclastogenesis (RANKL/RANK/OPG signaling pathway, estrogen/α-estrogen receptor). Pro-inflammatory cytokines (IL6, TNFα) and growth factors (TGF-β) produced by immune cells and adipose tissue also influence the balance between osteoblast and osteoclast formation and their apoptosis [28,29,30]. Genetic predisposition, oxidative stress, and cellular aging may also contribute to the development of osteoporosis [31,32,33].

### 3.1. Osteoclastogenesis

Osteoclasts develop from common precursors of the monocyte line of blood cells in the bone marrow; their differentiation is regulated mainly by monocyte-macrophage colony-stimulating factors, RANK ligands (M-CSF, RANKL/RANK/OPG), and estrogen [34]. Through the of the NF-kB transcription factor translocation, M-CSF and RANKL trigger a signaling pathway that promotes cell survival and differentiation into preosteoclasts and osteoclasts. This process involves multiple nuclear divisions, resulting in the formation of a large multinucleated cell firmly attached to the bone plates [35].

The RANK/RANKL/OPG system represents the central regulatory pathway responsible for osteoclast formation and activity. RANKL is a ligand produced by preosteoblasts, stromal cells, and activated T lymphocytes [36]. Upon binding, RANKL activates the receptor activator of NF-κB (RANK) on the surface of osteoclast precursors and triggers the differentiation and activation of osteoclasts, stimulating bone resorption processes [37]. In turn, osteoprotegerin (OPG) acts as a decoy receptor that binds with both membrane-bound and soluble RANKL, inhibiting RANK signaling activation and thereby limiting osteoclast formation [38]. OPG production strongly depends on estrogen stimulation [39]. Normally, estrogen is the main factor regulating the number of osteoclasts, triggering their apoptosis by interacting with α-receptors [40]. During menopause, estrogen deficiency decreases the OPG level with a relative increase in RANKL activity, which explains the sharp acceleration of bone loss in women after the age of 50 and the development of postmenopausal osteoporosis. At the same time, in osteoporosis, the RANK/RANKL ratio shifts towards RANKL, either due to increased RANKL levels or due to decreased OPG production, resulting in a higher bone resorption rate. 

The WNT pathway also takes place in osteoclasts but affects their regulation mainly indirectly through increased OPG expression in osteoblasts when the pathway is active, and increased RANKL expression when the WNT pathway is inhibited [41].

### 3.2. Osteoblastogenesis

Osteoblasts originate from mesenchymal stem cells in the bone marrow through their osteogenic differentiation. This dynamic process involves the sequentially initiated expression and synthesis of osteoblast-specific proteins and the decrease in cell proliferative ability [42]. The differentiation is regulated by various transcription factors and signal transduction [43]. Among them, the key role belongs to the transforming growth factor beta (TGFβ) group of proteins, which includes bone morphogenetic protein (BMP). Several studies have demonstrated that BMP proteins can stimulate mesenchymal stem cells to differentiate into osteoblasts, with BMP-9 having the strongest osteogenic inducing effect [44]. The TGFβ signaling pathway leads to the activation and nuclear translocation of the Smad2 transcription factor, enhancing the expression of the key intracellular transcription factor of osteoblasts, Runx2 [45]. The latter, in turn, triggers the expression of all specific proteins: type 1 collagen, RANKL, Osterix, and ALPL [46].

The Wnt signaling pathway governs osteoblast proliferation, differentiation, and migration [47]. Wnt pathway factors are a family of secreted glycoproteins, ligands for Frizzled (FZD) receptors. The pathway can be implemented through canonical and non-canonical pathways.

To activate the canonical pathway, WNT proteins (Wnt5a, Wnt3a) interact with LRP5/6 receptors on the cell surface with subsequent signal transduction within the cell, leading to the accumulation of β-catenin and its translocation into the nucleus [48]. As a signal transmitter, β-catenin activates the TCF transcription factor, which in turn triggers the expression of a large number of target genes responsible for bone formation. In osteoblasts, the target genes of the WNT pathway include Coll1A1, Osc, Osx, Opn, ALPL, and the BMP proteins. In the absence of WNT ligand binding, the degradation complex removes β-catenin, making the pathway inactive [49].

Alternatively, the β-catenin-independent non-canonical WNT pathway involves the ligands Wnt 4, Wnt5a, Wnt5b, Wnt6, Wnt7a, and Wnt11 [50]. Non-canonical WNT signaling occurs via two pathways: the Wnt/planar cell polarity (PCP) pathway and the WNT/calcium (Ca^2+^) pathway [48,51].

The canonical Wnt pathway mainly controls cell proliferation, including osteoblasts, while non-canonical pathways regulate cell polarity and migration.

Thus, WNT and BMP proteins regulate bone regeneration, influence the osteogenic differentiation of mesenchymal stem cells and osteoblast activity, modulate the expression of certain target genes (e.g., ALP), and also interact with each other and influence the expression of each other’s ligands in the overall bone remodeling system [45,52].

### 3.3. WNT Pathway Inhibitors

The natural inhibitors of the WNT pathway, sclerostin and DKK-1, block the pathway, thereby suppressing bone formation. These soluble proteins regulate the pathway activity by binding directly to WNT ligands (WIF, SFPR) or by competing with WNT ligands for binding to LRP5/6 receptors [53]. When the WNT pathway is inhibited, β-catenin undergoes ubiquitination and degradation, and new β-catenin is not formed, which disrupts signal transmission to the nucleus and prevents excessive cell division and degeneration.

Sclerostin is a secreted factor produced by osteocytes and osteoblasts in the late stages of development that blocks WNT signaling via the canonical and non-canonical pathways [54]. DKK-1 protein is produced by late osteoblasts and osteocytes. Unlike sclerostin, DKK-1 interacts with the LRP5/6 receptor via the clementin co-receptor and inhibits only the canonical WNT pathway [55]. Furthermore, it is not specific to bones but is also expressed in other tissues.

In the development of osteoporosis, an increased concentration of sclerostin and DKK-1 reduces the differentiation rate and activity of osteoblasts through the WNT pathway inhibition and results, for example, in reduced β-catenin and OPG levels in patients with postmenopausal osteoporosis [56]. Inhibition of the WNT pathway also reduces the OPG level in the RANK/RANKL/OPG system, thus stimulating the osteoclast differentiation.

An inactivation of sclerostin and DKK-1 increases the bone mass and bone density, which allows considering these WNT inhibitors as molecular targets for the treatment of degenerative bone diseases [57]. It was also proven that the genetic deletion of sclerostin enhances bone formation in mice and humans [58,59]. Inhibition or neutralization of DKK1 improves osteogenesis and canonical WNT pathway signaling activity [60] and enhances osteogenic differentiation [61].

### 3.4. Genetic Predisposition to Osteoporosis and Epigenetic Regulation

Although lifestyle and other acquired conditions are the main cause of osteoporosis, recent studies indicate a hereditary predisposition to reduced BMD, OP development of osteoporosis, or an increased bone fracture risk during life. The contribution of the genetic determinant to bone density varies from 30 to 85% of inherited gene variants [31]. However, in genome-wide GWAS (genome-wide associated) studies, the results are ambiguous—some 2006 studies did not show any dependence of BMD and bone fractures on loci and genes, while others identified many loci associated with fracture risk [62]. In addition to common polymorphisms and gene variations that cause a decrease in BMD and lead to the development of osteoporosis, there are also rare genetic variants that may contribute to a predisposition to osteoporosis at an early age, such as TNFRSF11, Col1a1, Col1a2, ESR1, LRP5, FONG, and ARHGEF3, but they are quite rare [63,64,65,66]. Mutations in some genes (X-chromosomal PLS3 mutation, non-synonymous substitution in BMP2) can also contribute, but they are rare or typical only for individual human populations [67,68]. However, the presence of these genetic predispositions, especially for hereditary fracture risks, plays a role in studies of people under the age of 65, after which the contribution of heredity decreases sharply due to age-related physiological changes in bone tissue and lifestyle [69].

Epigenetic factors, such as DNA methylation and histone modifications, miRNAs and other non-coding small RNAs also impact the predisposition to develop low BMD and osteoporosis. DNA methylation in genes related to bone metabolism (SOST, Era, Runx2, RANK, RANKL, OPG, and Wnt proteins) correlates negatively with the presence of osteoporotic hip fractures. Among histone modifications, methylation is usually associated with the initiation of gene expression in methylated regions. Therefore, methylation enhances osteoclast differentiation, but in osteoblasts, it all depends on the methylation site [70]. The epigenetic mechanism is also critical for regulating osteoclast differentiation [71]. Acetylation of histones at the gene promoter sites during differentiation induces the formation of osteoclasts, while deacetylases inhibit osteoclast differentiation. In particular, Sirtuin 1 (SIRT1), a class 3 deacetylase, acts as a protein of the cellular stress response, suppresses the Nf-kB signaling pathway in osteoclast formation, and also stimulates the development of osteoblasts [68].

MicroRNAs, a class of small non-coding RNAs that regulate gene expression through different mechanisms, also act as a part of epigenetic machinery [72]. There are a number of microRNAs that provide the maintenance of bone homeostasis, influencing osteoblastogenesis and osteoclastogenesis [73]. There is evidence that microRNAs can regulate the work of deacetylases in the control of osteoclast differentiation, creating a complex multi-step regulatory network. Studies show a significant correlation between microRNAs and bone metabolism, and disruption of their expression is associated with impaired bone metabolism and osteoporosis [74]. Numerous studies indicate that microRNAs can induce osteoblast differentiation, which leads to successful bone reconstruction and prevents the progression of osteoporosis. For example, miR-96 affects the activity of the SOST protein, ALP activity, and mineralization [75]. MicroRNAs can guide bone marrow-derived mesenchymal stromal cell (BMSC) specialization by influencing gene expression and signaling pathways involved in bone formation, thus representing possible candidates for determining the pathogenesis of osteoporosis and for OP treatment.

Long non-coding RNAs (lncRNAs) represent another key element that participates in the regulation of skeletal aging and mesenchymal stromal cell (MSC) function. Although the precise molecular mechanisms and particular lncRNAs that influence MSCs remain to be fully elucidated, several candidates have already been identified. A striking example is lncRNA-MEG3, which has been shown to enhance the osteogenic differentiation of MSCs, potentially counteracting age-related bone loss [76].

### 3.5. Osteoimmune Interactions and Regulation: A Delicate Balance

Bone remodeling is also regulated by a complex network of interactions between the immune system and bone tissue through shared cytokines, receptors, and regulatory pathways. The discovery of immune cell-regulated osteoclast activation through the RANKL/RANK/OPG system stimulated the emergence of osteoimmunology as a distinct research field [77]. Under physiological conditions, immune cells participate in maintaining bone homeostasis by responding to microdamage and stimulating osteoclast activity to break down old bone tissue.

Several lymphocyte populations contribute to the maintenance of bone remodeling [78]. There are numerous connections between T cells and bone cells, as almost all T cell populations can influence bone cells, primarily osteoclasts. Thus, activated T cells and B cells produce cytokines and RANKL, which leads to osteoclast activation [79]. Specifically, Th1 cells produce TNF-α and IL-1, which stimulate bone resorption. However, Th2 cells produce anti-inflammatory cytokines (IL-10 and IL-4), which inhibit osteoclastogenesis.

Th17 cells are also involved in regulating the RANKL/RANK/OPG pathway. The authors demonstrated the role of the Th17 subset in regulating local osteoclastic activation in rheumatoid arthritis [80]. The IL-17 they produce can stimulate cells that support osteoclastogenesis, such as synovial fibroblasts and osteoblasts, to express RANKL and increase bone resorption. Furthermore, Th17 cells can enhance RANKL expression by inducing the production of inflammatory factors (TNF-α, IL-1, and IL-6) [81].

Regulatory T cells (Treg, FoxP3+) suppress osteoclastogenesis through IL-10, TGF-β, and IL-4 secretion, or by the contact interaction of CTLA-4 with CD80/86 on osteoclasts, which induces their apoptosis [82].

B cells produce proinflammatory cytokines IL-6, TNF-α, and RANKL, which induce osteoclastogenesis [29]. However, B lymphocytes also contribute to the suppression of bone resorption through the production of OPG. Macrophages and monocytes, as osteoclast precursors, also produce proinflammatory cytokines (IL-1, IL-6, TNF-α), which in low concentrations modulate physiological bone remodeling [83].

However, in osteoporosis, especially in postmenopausal and inflammatory types, a shift towards a chronic immune response provides a predominance of bone resorption over formation. As women age, postmenopausal osteoporosis often develops due to estrogen deficiency. Estrogen is known to have a direct immunomodulatory effect [84]. Its deficiency triggers a series of changes in immune system cells, including the activation and hyperproliferation of T cells and a shift in their cytokine profile. Increased production of TNF-α directly stimulates osteoclastogenesis and potentiates the RANKL signal, thereby lowering the activation threshold for progenitor cells. Additionally, TNF-α induces RANKL expression on both stromal cells and osteoblasts [85]. Estrogen deficiency increases the number of Th17 cells, which produce IL-17A, leading to the stimulation of osteoblasts and stromal cells and an increase in RANKL expression. Osteoporosis is characterized by Treg dysfunction and decreased suppressive activity, which leads to the release of the “brake” on osteoclastogenesis [86]. It is known that estrogen has a stimulating effect on osteoblasts, increasing the production of OPG, but with its deficiency, there is a decrease in OPG production, which subsequently leads to the relative dominance of RANKL [39].

Furthermore, aging processes in the body are associated with chronic, low-grade systemic inflammation—“inflammaging”. Osteoporosis and aging are accompanied by an accumulation of senescent cells (osteoblasts, osteocytes, and immune cells), producing pro-inflammatory cytokines (IL-6, IL-1β, and TNF-α), chemokines, and proteases and creating a pro-resorptive background [87]. A shift towards the production of pro-inflammatory cytokines by immune system cells and the suppression of Treg cell activity lead to accelerated bone resorption. 

Understanding the mechanisms of the osteoimmune axis opens new possibilities for OP therapy. The development of novel treatment options should target multiple key pathways: inhibition of RANKL, TNF-α, and IL-6 to suppress their pro-resorptive and pro-inflammatory effects; Th17 blockade to suppress a potent stimulus of osteoclastogenesis; modulation of signaling pathways in mature osteoclasts to reduce their activity; and stimulation of Treg cells.

### 3.6. Oxidative Stress and Cellular Senescence

With age, reactive oxygen species (ROS) accumulate in the body, leading to an imbalance in the pro- and antioxidant systems. Oxidative stress brings catastrophic consequences for bone marrow-derived MSCs. Suppression of proliferation and differentiation: high levels of ROS directly damage the DNA, lipids, and proteins of MSCs, inhibiting their ability to divide and differentiate into osteoblasts (bone-building cells). Oxidative stress often switches the differentiation potential of MSCs from the osteogenic pathway to the adipogenic pathway. As a result, instead of forming bone tissue, adipose tissue accumulates in the bone marrow, which is a characteristic feature of osteoporosis. ROS are one of the main triggers for MSCs to enter a state of cellular senescence [32].

Cellular senescence is a state of irreversible cell cycle arrest entered by MSCs under the influence of stress factors, including oxidative stress. The main danger of senescent MSCs is not only the loss of regenerative function but also the secretion of proinflammatory cytokines, chemokines, and proteases, the so-called “senescence-associated secretory phenomenon” (SASP). As a result, chronic low-grade inflammation creates a proinflammatory bone microenvironment and further suppresses healthy MSCs and osteoblasts while simultaneously activating osteoclasts. Therefore, SASP disrupts the delicate balance of bone remodeling, favoring resorption [33].

Changes in the secretome—the complex of substances secreted by cells—also significantly contribute to skeletal aging. It has been shown that differences in the secretomes of aged and young BMSCs can contribute to the development of osteoporosis [88].

Therapy using extracellular vesicles (EVs) is a promising approach to combating age-related changes. The anti-aging potential is not limited to a single EV source: EVs from other stem cell types also exhibit similar protective effects. In particular, EVs isolated from umbilical cord MSCs contain a rich set of anti-aging signals and have shown the ability to rejuvenate senescent BMSCs derived from adults [89].

### 3.7. The Relationship Between Bone and Adipose Tissue: Regulatory Pathways of Osteogenesis and Adipogenesis in Osteoporosis

Bone tissue is now recognized as a dynamic, metabolically active organ. The function of adipose tissue within the bone marrow was long considered to be merely the space filling between cells. Recent research has demonstrated a close functional link between osteogenesis and adipogenesis [30]. Bone and adipose tissue cells share a common origin. Osteoclasts originate from hematopoietic stem cells and share a common lineage with monocytes and macrophages. MSCs are the precursors of osteoblasts. Bone marrow MSCs are multipotent and can differentiate into osteoblasts, adipocytes, chondrocytes, and other cell types [90]. The differentiation of MSCs is strictly regulated by a complex network of transcription factors and signaling pathways. 

The key factor of osteogenic differentiation, Runx2/Cbfa1, activates osteocalcin and type I collagen genes in osteoblastic phenotype cells [91]. The Wnt/β-catenin signaling pathway plays a crucial pro-osteogenic role as the main pathway regulating differentiation in cells throughout the body. PPARγ (peroxisome proliferator-activated receptor gamma) is the primary regulator of adipogenic differentiation in MSCs. Its activation triggers a cascade of genes that ultimately lead to the formation of a mature adipocyte [92]. Activation of PPARγ stimulates adipocyte formation and simultaneously suppresses osteogenic differentiation. It is important to note that the osteo- and adipogenesis pathways are well-balanced through their mutual regulation. For example, the factors produced by adipocytes inhibit osteogenesis by blocking BMP signaling through a mechanism mediated by adipokine-induced activation of the NF-κB pathway [93]. At the same time, osteoblast-secreted factors, such as IL-11, suppress adipogenesis [94].

In osteoporosis, this delicate balance shifts in favor of adipogenesis. The number and activity of osteoblasts decrease, while the volume of adipose tissue in the bone marrow increases significantly. These changes arise due to the aging process in the body under the influence of pathological factors (estrogen deficiency, chronic inflammation), which also provide a loss in the proliferative and differentiating potential of MSCs in the osteogenic direction.

All of these processes are associated with the dysregulation of key signaling pathways. First and foremost, increased levels of endogenous inhibitors (DKK-1, sclerostin) suppress the Wnt/β-catenin pathway in osteoblasts, providing the activation of PPARγ. Several factors, such as fatty acids and proinflammatory mediators, can hyperactivate PPARγ, which directly promotes the differentiation of mesenchymal stem cells into adipocytes [95]. Bone marrow adipocytes, like mesenchymal stem cells, are able to express RANKL, stimulating osteoclasts. As a result, the pro-resorptive signal predominates, leading to bone loss. 

A significant role in regulating bone tissue remodeling in osteoporosis belongs to adipokines, cytokines secreted by adipocytes when adipogenesis dominates over osteogenesis. Adipose tissue is an active endocrine organ that secretes adipokines [96].

Thus, leptin has dual effects. Systemically, through the hypothalamus, it can have a pro-osteogenic effect. However, locally in the bone marrow, leptin stimulates osteoblastogenesis but can also enhance resorption [97]. Normally, adiponectin supports proliferation, migration, and mineralization in osteoblasts and at the same time limits the functional activity of osteoclasts. Studies have shown a pro-osteogenic role for adiponectin in in vivo and in vitro models, with increased osteoblast differentiation and activity, along with lower levels of osteoclastogenesis [98].

Visfatin also has an effect on bone tissue. It stimulates the production of proinflammatory cytokines (IL-6, TNF-α), which in turn activate osteoclastogenesis mediated through Toll-like receptor 4 (TLR4) [99].

These negative factors lead to bone imbalance in osteoporosis. Understanding the relationship between osteogenesis and adipogenesis opens up new targets for osteoporosis therapy. Key targets may include PPARγ inhibitors, Wnt pathway activators, and adipokine-related targets.

Thus, the interaction between bone and adipose tissue represents a complex, regulated system. In osteoporosis, this regulatory system is thrown out of balance by various molecular disturbances, leading to adipogenic differentiation of MSCs at the expense of osteogenesis. A shift in cell differentiation, as well as changes in the secretory profile of adipocytes and chronic inflammation, create conditions for increased bone resorption and consequently bone loss.

## 4. Therapeutic Approaches Used in Routine Practice

The main goal of osteoporosis treatment is to reduce the risk of fractures. Therefore, the treatment efficacy is assessed by the decrease in the fracture frequency, also using additional markers such as an increase in bone mineral density (which correlates particularly with the frequency of femoral bone fractures), levels of bone remodeling markers [25], etc. Taking into account the mechanisms of bone tissue renewal, two classes of agents have been proposed to treat osteoporosis: antiresorptive drugs that target osteoclasts and inhibit bone resorption and destruction, and anabolic agents that act on osteoblasts and stimulate osteogenesis and restoration of bone structure and bone mass [10,100]. In Table 1, we summarize the information on drugs used in real-world clinical practice for the treatment of osteoporosis, as well as targeted agents under development.

### 4.1. Anti-Resorptive Agents

A fairly extensive list of drugs have proven antiresorptive effects: bisphosphonates, calcitonin, hormone therapy, denosumab, etc. [10,137,138,139]. Below, we describe the main representatives of the class.

#### 4.1.1. Bisphosphonates

Traditionally, bisphosphonates represent the first line of OP treatment (except ibandronic acid), unless there are special circumstances. Their clinical use began in 1995, after the approval of alendronate [140]. Bisphosphonates affect all parts of the skeleton, so they also find application for the treatment of other diseases accompanied by excessive bone damage, such as metastases, Paget’s disease, etc.

**Figure 3:**
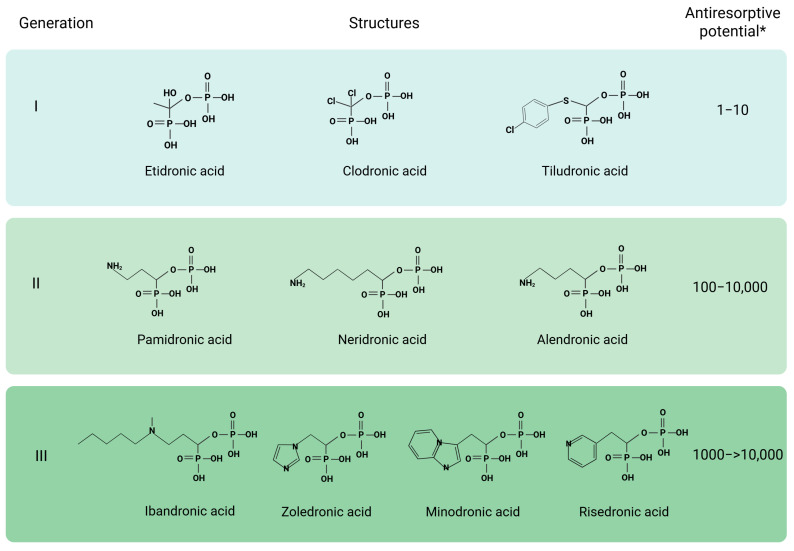
Chemical structures and antiresorptive potential of different generations of bisphosphonates. * Antiresorptive potential relative to nitrogen-free bisphosphonates.

By their chemical nature, bisphosphonates are derivatives of inorganic pyrophosphate (Figure 3). They bind tightly to hydroxyapatite crystals and inhibit their destruction, thus shifting the balance towards osteogenesis and providing bone mass acquisition. This process occurs most rapidly in areas of active bone remodeling. On the other hand, bisphosphonates can inhibit calcification, which negatively affects bone formation. The severity of a particular effect depends on the generation of the drug; each subsequent generation, due to certain chemical modifications, acquires a significantly improved ability to suppress bone resorption without affecting calcification. Namely, the introduction of nitrogen increases the antiresorptive efficacy of nitrogen-containing bisphosphonates by 10–10,000 times compared to nitrogen-free ones. The ranking of the binding capacity of bisphosphonates is as follows: zoledronate > alendronate > ibandronate > risedronate [141].

Moreover, bisphosphonates possess a suppressive effect on osteoclasts. Simple first-generation bisphosphonates (nitrogen-free) are metabolized in osteoclasts into toxic ATP analogues, thus causing cell death. Nitrogen-containing bisphosphonates of the next generations promote osteoclast apoptosis by inhibiting farnesyl pyrophosphate synthase (FPPS) with the subsequent loss of long-chain isoprenoid lipids required for the prenylation of G proteins (Ras, Rho, Rab, etc.), signaling proteins necessary for the survival and activity of osteoclasts. Inhibition of FPPS also leads to the formation of another endogenous ATP analogue (ApppI), which inhibits mitochondrial adenine nucleotide translocase, causing osteoclast apoptosis [101]. Third-generation bisphosphonates combine greater antiresorptive activity and affinity for hydroxyapatite with a longer duration of action and less frequent dosing, which increases therapy adherence.

Alendronate, zoledronic acid, and risedronate acid increase the BMD values and reduce the risk of osteoporotic fractures in men, postmenopausal women, and patients with glucocorticoid-induced osteoporosis [1]. Ibandronate acid, with proven efficacy in preventing vertebral fractures, has not been shown to reduce the risk of hip fractures.

Oral bisphosphonates require strict adherence to the dosage regimen because of the low bioavailability of this form of the drug and possible adverse effects on the gastrointestinal tract (heartburn, indigestion, esophageal erosion, and ulcers). Parenteral administration of bisphosphonates may be accompanied by flu-like symptoms (fever, muscle pain, and arthralgia). This unpleasant but non-fatal complication is usually well-controlled by paracetamol. Before starting bisphosphonate therapy, it is recommended to take calcium and vitamin D to prevent hypocalcemia [142].

Unbound bisphosphonates are excreted by the kidneys, so a significant reduction in renal function becomes the main limitation to their use. The borderline level of estimated glomerular filtration rate that precludes the use of bisphosphonates is 30–35 mL/min (<35 for alendronate and zoledronic acid; <30 for risedronate and ibandronate).

The optimal duration of bisphosphonate therapy has not been stated. Long-term use of bisphosphonates can lead to the opposite effect: excessive bone fragility. Animal studies have shown ambiguous results in assessing the bone mechanical properties during bisphosphonate therapy and revealed increased microdamage accumulation and a deterioration in mechanical properties. However, despite a slight increase in the number of microdamages during prolonged observation, bone strength continued to decrease, which may point to the absence of a direct association between the bone functional state and the number of microdamages. Since this effect of bisphosphonates was studied at doses exceeding therapeutic levels, the results cannot be translated into clinical practice [143,144,145]. An increased risk of low-energy femur fractures has been reported with bisphosphonate use. In 2010, the FDA reported that it remains unclear whether this effect is a consequence of bisphosphonate therapy but did not rule out the possibility that it could develop with their long-term use. Invasive dental procedures in patients receiving long-term bisphosphonate therapy may lead to a formidable but rare complication known as jaw osteonecrosis, characterized by exposed and necrotic bone in the maxillofacial region not healing within eight weeks. Taking into account the possible adverse effects of long-term use, the accumulation of bisphosphonates in bone tissue, and their subsequent release into the bloodstream, it is recommended to consider the possibility of a “drug holiday” when the risk outweighs the benefit. For low and moderate fracture risk, the question of a medication holiday arises after 3–5 years of medication or three consecutive annual administrations of zoledronic acid; for high risk, after 6–10 years of taking oral bisphosphonates and six annual administrations of zoledronic acid. The duration of the break is not defined, and bisphosphonate administration may be resumed in the case of a fracture, a significant decrease in BMD, and an increase in levels of bone remodeling markers. Given the effect of bisphosphonates on bone remodeling, their negative impact on fracture healing has been discussed. In animal models, bisphosphonates improved the mechanical properties of callus but increased the callus size and delayed its remodeling, while the fracture healing time was not prolonged [146,147].

Taking into account the potential teratogenicity and placental permeability of bisphosphonates, their use is not recommended for pregnant women or women of childbearing potential without consulting a specialist [142].

#### 4.1.2. Strontium Ranelate

Strontium ranelate is a salt of ranelic acid that provides a significant bone mass increase due to Sr^2+^ ions, similar in its physical and chemical properties to Ca^2+^, the essential element of the bone mineral part. Strontium affects bone tissue cells through the Ca-sensitive receptor (CaSR) and can also be incorporated into bone cells, increasing their density and reducing the risks of osteopenia and osteoporosis. By acting on osteoblasts, strontium ranelate increases their proliferation and differentiation, as evidenced by increased expression of osteogenic genes such as Runx, ALP, BSP, and BGP, enhances the synthesis of bone matrix proteins, and suppresses osteoblast apoptosis. At the same time, it suppresses the formation and differentiation of osteoclasts and promotes their apoptosis, thus decreasing bone resorption [103,148]. Strontium ranelate is not metabolized in the body and is excreted mainly through the kidneys.

According to clinical guidelines, strontium ranelate is not recommended for routine use in the treatment of osteoporosis due to adverse effects, including severe skin reactions such as toxic epidermal necrolysis, cardiovascular disease (myocardial infarction), and thromboembolic complications. Intravenous administration of high doses of Sr^2+^ results in hypocalcemia caused by increased renal Ca^2+^ excretion [103]. In 2017, the drug was discontinued, but it remains available in a number of countries. Its use is considered as a second-line drug only when other anti-OP drugs are either unavailable or poorly tolerated by the patient [106]. 

Modified biomaterials with strontium ions could be especially useful in healing defects caused by osteoporotic fractures, as their studies show a significant increase in bone formation at the implant border with increased osteoblast activity and decreased osteoclast activity. In particular, iron oxide nanospheres-hydroxyapatite-strontium@collagen (IONSs-HA-SR@C) have demonstrated antibacterial activity and significant efficacy in treating infections. Furthermore, these materials possess sufficient biocompatibility [103].

#### 4.1.3. Calcitonin

Calcitonin, one of the first drugs for postmenopausal OP treatment, is a 32-amino acid peptide produced by parafollicular cells of the thyroid gland [149]. By acting on osteoclast receptors, it causes their outflow from areas of active bone resorption, and by affecting osteoblasts, it stimulates bone formation. Calcitonin has been shown to reduce solely the risk of vertebral fractures and only when administered nasally. Compared to other drugs, it has not shown any advantages and remains in the arsenal of OP treatment as a second-line drug thanks to its possible association with malignant processes. Calcitonin is preferable in some cases because of its analgesic effect, which is especially recommended for certain conditions, such as neuropathic pain, osteoporotic vertebral fractures, and Paget’s disease [106,107].

#### 4.1.4. Estrogen-Progestin Therapy

The amount of estrogen, which provides osteoclast apoptosis and plays a protective role, decreases during menopause. Estrogen-progestin therapy (EPT) compensates for the hormone deficiency and reduces bone loss by suppressing osteoclast activity [110]. EPT uses oral conjugated equine estrogen (oCEE) and medroxyprogesterone acetate (MPA). Women who need hormone therapy after a hysterectomy are recommended to take estrogen alone, but those with an intact uterus need to take combined progestin + estrogen treatment to avoid negative effects on the endometrium [10]. Both oCEE + MPA and oCEE alone significantly reduce the risks of hip and vertebral fractures to an extent comparable to that of bisphosphonate therapy [111]. However, these benefits may disappear over time after therapy discontinuation, requiring physicians to switch to another drug at the end of EPT [150]. EPT in post/perimenopause is approved for the prevention of bone loss in postmenopause but is not a first-line therapy due to possible adverse events (high risk of cardiovascular disease, stroke, venous thromboembolism, and invasive breast cancer), and the health risks outweighing the benefits. It is debated, though, that the risks of using EPT in healthy women under the age of 60 or within ten years of menopause are low. Their use is permitted when non-estrogenic treatments are considered inappropriate [151].

#### 4.1.5. Selective Estrogen Receptor Modulators (SERMs)

This group of drugs, also known as estrogen agonists/antagonists, includes raloxifene, conjugated estrogens/bazedoxifen, and lasofoxifene. Drugs of this type bind to estrogen receptors and exhibit selective estrogenic activity depending on the cell or tissue type [10].

Raloxifene is a second-generation selective estrogen receptor modulator that exerts an estrogen-agonistic effect on bone tissue receptors. Its mechanism of action engages binding to estrogen receptors, which leads to the activation of estrogen pathways (estrogen-agonistic effect) and blockade (estrogen-antagonistic effect) in tissues where estrogen receptors are present. Raloxifene inhibits accelerated bone resorption in both the short- and long-term, increasing BMD and bone strength [114]. Unlike tamoxifen (a first-generation selective estrogen receptor modulator), which has an estrogen-like effect on the uterus, raloxifene has an estrogen-antagonistic effect on the uterus and breast. It is used to treat postmenopausal osteoporosis and reduce the risk of invasive breast cancer in postmenopausal women. In healthy postmenopausal women, raloxifene prevents bone loss and reduces the risk of spinal fractures, but not fractures in other locations. It can also be used as a weaker antiresorptive therapy for high-risk patients during a break in bisphosphonate treatment [1]. There is evidence of its efficacy in increasing BMD in the lumbar spine in postmenopausal women with end-stage renal failure [152]. Undesirable raloxifene effects include thromboembolic complications (deep vein thrombosis, pulmonary embolism), vaginal bleeding, stroke, and cardiovascular disease, which require careful monitoring. The number of raloxifene prescriptions remains low because of possible adverse effects and the availability of safer and more effective alternatives [115]. New third-generation drugs are being developed, including lasofoxifene, bazedoxifene, osemifene, and arzoxifene [153]. The combination of conjugated estrogens with bazedoxifene (conjugated estrogens/bazedoxifen) significantly reduces the risk of vertebral and non-vertebral fractures. The drug not only increased BMD in the hip and spine in postmenopausal women compared to the placebo but also reduced hot flashes. Lazoxifene lowered the relative risk of vertebral fractures and other fractures by 42% and 24%, respectively. Despite these positive aspects, this group of drugs is not considered as a first-line OP therapy due to the increased risk of venous thromboembolism, as well as the risk of developing breast cancer and stroke during long-term therapy [1,154].

#### 4.1.6. Denosumab

Denosumab, a fully human monoclonal antibody (IgG2) generated by recombinant DNA technology, is one of the most studied biologics and the first drug of the class that entered clinical practice for OP treatment. It is a full-length antibody (~150 kDa) without Fc fragment modification. The genes encoding the heavy and light chains of the antibody are derived from RANKL-specific human B-lymphocytes, inserted into expression vectors, and cloned in cell lines (most often CHO). Denosumab blocks RANKL, an essential factor regulating osteoclast activity [116].

Denosumab is administered at a dose of 60 mg subcutaneously once every 6 months. Its efficacy has been confirmed in numerous randomized controlled trials demonstrating a 30–50% reduction in the risk of hip and vertebral fractures with long-term use [117]. Denosumab provides an increase in BMD, with a greater effect in patients with lower baseline bone density. Previous OP treatment also influences denosumab therapy, as demonstrated in a Chinese population of women. The use of zoledronic acid significantly reduced BMD gain; alendronic acid and raloxifene had a lesser effect, while teriparatide had no effect at all [155]. However, the use of denosumab after bisphosphonates still provides a positive effect on BMD in the lumbar spine in postmenopausal women compared to continuous bisphosphonate therapy [156].

The drug is well-tolerated, and its use is not accompanied by serious adverse events. Denosumab treatment may lead to hypocalcemia, especially in patients with a decline in renal function (<30 mL/min). Therefore, it is recommended to maintain optimal blood calcium levels, particularly in patients with severe renal failure and those on hemodialysis [157].

During denosumab therapy, temporary discontinuation is not recommended as it has been associated with a BMD decrease and an increased risk of vertebral fractures. Follow-up treatment with alendronate or zoledronic acid, but not risedronate, slowed the bone loss caused by denosumab discontinuation [158,159]. However, after denosumab discontinuation in some patients with increased CTX levels, even two infusions of zoledronic acid at 6-month intervals did not prevent bone loss, which raises the question of a personalized approach to optimizing therapy [160].

### 4.2. Anabolic Agents

The group of anabolic agents includes teriparatide, abaloparatide, and romosozumab. Their mechanism of action is based on increasing bone formation to a greater extent than the current level of resorption. Teriparatide and abaloparatide, PTH receptor 1 agonists, primarily stimulate bone formation based on remodeling, while romosozumab primarily stimulates bone formation based on modeling [161]. In line with new trends, for patients at very high fracture risk, it is advised to consider starting OP therapy with anabolic drugs (or a combination with antiresorptive drugs), as the standard start with bisphosphonates or denosumab does not always provide sufficient efficacy in this group [9,16,162,163]. Moreover, there is evidence in favor of initiating OP therapy with anabolic drugs regardless of fracture risk [100]. In turn, after completing the treatment with anabolic drugs, it is recommended to prescribe bisphosphonates (if the target T-score values are close to being achieved) or denosumab, as it increases BMD after anabolic therapy better than bisphosphonates [163,164].

#### 4.2.1. Teriparatide and Abaloparatide

Teriparatide and abaloparatide are osteoanabolic drugs similar in action to the biological effects of parathyroid hormone (PTH) and 84-amino acid polypeptide, which activate PTH1 receptors in the kidneys and bones, regulating calcium metabolism. PTH directly stimulates osteoblasts and osteocytes, promotes their osteoanabolism and the differentiation of stem cells into osteoblasts, increases the activity of osteoblasts and extends their lifespan. PTH also reduces the sclerostin expression, further supporting bone formation. Otherwise, PTH stimulates the RANKL production, increasing osteoclast activity and bone resorption [165].

Teriparatide, the first anabolic agent approved for OP treatment, represents a recombinant active 34-amino acid N-terminal fragment of human PTH. Teriparatide increases BMD mainly in trabecular bone (lumbar spine), and to a lesser extent in cortical bone (femur), with even a slight loss of BMD observed in patients with osteoporosis in the first months of treatment. It is used in patients with previous low-energy fractures or in individuals at high fracture risk, as well as in those who cannot take oral therapy. Teriparatide may also be used in high-risk patients during a bisphosphonate holiday [1].

Abaloparatide (ABL) is approved by the FDA for the treatment of postmenopausal women. Compared to teriparatide, ABL demonstrates a selective preference for the G protein-dependent (GTPγS-sensitive) conformation of the PTH receptor (RG), which leads to shorter intracellular activity and decreased calcemic response and RANKL production. This, in turn, stimulates resorption to a lesser extent and has a beneficial effect on cortical bone tissue. Thus, abaloparatide provides a longer prevalence of bone formation over resorption, giving a more pronounced net anabolic effect than teriparatide [126,166]. The ACTIVE study confirmed an effect of ABL on increasing BMD in the lumbar spine similar to that of teriparatide, but abaloparatide showed a significantly greater increase in BMD in the femoral neck (+3.60% vs. +2.66%) and in the femur as a whole (+4.18% vs. +3.26%), indicating a greater benefit of ABL for cortical bone [167]. In model studies, both analogues demonstrated improvement in bone microarchitecture [168] and increased TBS, a surrogate marker of bone quality. Their possible adverse effects include orthostatic hypotension, hypercalcemia, and urolithiasis.

The use of parathyroid hormone analogues for more than two years is not recommended, as animal models have demonstrated a dose-dependent increase in the risk of osteosarcoma formation [14]. It remains unknown whether these drugs can cause osteosarcoma in humans, so they are not advised for patients with an increased osteosarcoma risk, including those with Paget’s disease, with unexplained increases in ALP levels, with open bone epiphyses, bone metastases or skeletal system tumors, hereditary diseases predisposing to the development of osteosarcoma, or a history of radiation therapy to the skeletal region.

#### 4.2.2. Romosozumab

Over the past decade, attention has grown to the strategy of targeting WNT pathway regulators (primarily sclerostin and DKK-1) for the treatment of diseases associated with bone metabolism imbalance [45]. In 2019, the first and only monoclonal antibody-based drug, Evenity (romosozumab), a sclerostin inhibitor, was approved for OP treatment [118]. The drug has demonstrated efficacy in the treatment of postmenopausal osteoporosis, providing increased bone formation and reduced bone resorption. Romosozumab keeps its efficacy even when re-administered after therapy with teriparatide, bisphosphonates, and denosumab [169]. The introduction of the drug into clinical practice was limited by an increased cardiovascular risk revealed in clinical studies [15], although this effect was subsequently refuted [170].

The efficacy of anabolic drugs in preventing low-energy fractures has been widely proven [165], and the probability of serious side effects (including the risk of developing malignant neoplasms for teriparatide and cardiovascular disease for romosozumab) is gradually decreasing, although their long-term safety requires further analysis. Their widespread use is also limited by the high cost, which should be taken into account in real clinical practice [100,171].

Currently used drugs have a positive effect by increasing bone density and reducing the fracture risk. However, their range is quite limited, and they possess certain drawbacks, such as undesirable effects and restrictions associated with comorbidity. Various obstacles to their prescription require the search for new OP treatment strategies. The possibility of targeting the pathways underlying bone metabolism could significantly improve therapeutic options and provide novel, powerful tools in the fight against osteoporosis.

## 5. Prospects for Targeted Therapy

### 5.1. Agents at the Clinical Trial Stage

The first and second stages of clinical trials mainly focus on new molecules capable of variable impact on osteoclast activity and the stimulation of osteoblasts. Namely, the ongoing development of monoclonal antibodies, in particular RANKL-blocking ones, seems to be one of the most promising areas in the treatment of osteoporosis [113]. These drugs are already demonstrating high efficacy in reducing the fracture risks, and their safety profiles meet the requirements for long-term use in medical practice.

A very promising direction deals with novel drugs directed to signaling pathways regulating bone degradation. This group includes anti-TGF-β (fresulimumab) and anti-IL-6 receptor (sarilumab) antibodies [172,173,174]. Blosozumab, a synthetic anti-sclerostin IgG-type monoclonal antibody structurally different from romosozumab, has shown its efficacy in a phase II study [130].

Phase II and III clinical trials are also in progress for co-formulated medicines combining biological drugs with traditional bisphosphonates or new small molecules, such as denosumab + teriparatide or romosozumab + zoledronic acid [175]. Combined use demonstrated a more pronounced reduction in bone loss in preliminary clinical trials.

Phase III clinical trials are now ongoing for cathepsin K inhibitors, such as odanakatib, balicatib, and ONO-5334 [176]. Large multicenter studies began in 2024, involving several thousand patients at high risk of fractures due to osteoporosis. These investigations would allow for obtaining more precise data on long-term safety and efficacy. For example, preliminary analysis results showed that new anti-cathepsin K compounds reduced the risk of hip and spine fractures by 30–40% compared to existing drugs. However, due to the high risk of stroke in patients, odanacatib has still not undergone clinical trials and requires additional research [121].

Bruton’s tyrosine kinase (BTK) inhibitor (Tirabrutinib) and MAPK kinase inhibitors (BMS-582949) have demonstrated potential therapeutic effect for OP treatment due to the inhibition of excessive osteoclastic activity [124,125].

In general, modern approaches to targeted OP therapy for osteoporosis involve not solely the design and creation of new molecules but also the integration of personalized methods and the most precise drug delivery, which promises to significantly improve the treatment efficacy and the life quality of patients. Future implementation of these innovations in clinical practice would bring a transition to more accurate and safer therapy based on the molecular and genetic characteristics of each patient [177].

### 5.2. Key Trends in the Development of Novel Approaches for Targeted Osteoporosis Therapy

Over the past 5 years, the development of OP therapy by means of monoclonal antibodies has remained one of the most active fields of research. Their high selectivity for molecular targets associated with pathological bone remodeling processes significantly reduces the risk of fractures while minimizing systemic side effects.

Current research focuses on creating new antibody variants with improved pharmacokinetic characteristics, such as lower immunogenic activity or a longer half-life [178]. This allows for reducing the dose frequency, increasing patient comfort, and mitigating the risk of immune reactions. In particular, novel denosumab variants with modified Fc fragments provide more effective circulation in the blood and sustained suppression of osteoclast activity.

Preclinical trials are also actively ongoing for antibodies targeting other bone remodeling-associated molecular targets. In particular, the targeted inhibition of sclerostin, a Wnt pathway antagonist, opens up new possibilities for stimulating osteoblastic activity. The anti-sclerostin antibody BHQ880 is a monoclonal antibody (IgG4) obtained from a mouse hybridoma and then humanized (approximately 90% of the sequences replaced with human ones). BHQ880 blocks sclerostin, thus activating the WNT/β-catenin pathway and enhancing osteoblast differentiation and bone formation [179]. Clinical trials have already demonstrated the effectiveness of such antibodies in increasing bone mass and reducing the risk of fractures [180]. Wnt pathway activation can also be implemented by inhibiting DKK-1 protein [181]. Currently, a number of potential therapeutic drugs aimed at blocking DKK-1 have been proposed, the most promising of which are specific antibodies [132,182]. 

It should be noted that therapy with anti-sclerostin antibodies leads to a compensatory increase in DKK1 level. This phenomenon stimulated the studies of simultaneous DKK1 and sclerostin inhibition with bispecific antibodies that demonstrated a positive effect [183]. In turn, DKK1 inhibition not only improved impaired osteoblastogenesis but also reduced TNF-induced sclerostin expression in a mouse model [184].

Bispecific antibodies have recently gained popularity in the field of targeted anti-OP therapy. We should mention here the creation of bispecific antibodies for double blocking RANKL and sclerostin, or sclerostin and DKK1, which allows for both the suppression of osteoclastic activity and stimulation of osteoblastic function [131]. Amgen, Novartis, and UCB Pharma are investigating similar formats (but there are no data on approved drugs yet). Targeted therapeutics of this class can significantly increase the treatment efficacy and reduce the need for the long-term use of several separate drugs [183].

Overall, the development of antibodies in the treatment of osteoporosis shows significant progress, providing a more precise and safer effect on the pathological mechanisms of the disease. Broadening of antibody functionality and combining them with other therapeutic approaches promises to expand the range of treatment strategies and improve the life quality of patients at high fracture risk [185].

Special attention is paid to the development of personalized medicine based on pharmacogenetic research. The identification of genetic markers that predict therapy response makes it possible to create individualized treatment protocols for achieving optimal clinical outcomes [186,187]. For instance, polymorphisms (FokI, BsmI) in the VDR gene (vitamin D receptor) affect vitamin D metabolism and sensitivity to vitamin D preparations [188,189]. Patients with the F (FokI) allele respond better to therapy with active forms of vitamin D (calcitriol, alfacalcidol). In the case of the COL1A1 gene (type I collagen), the Sp1 polymorphism is associated with an increased risk of fractures and impaired bone formation [190]. Carriers of this variant respond less well to standard bisphosphonates but may show a better response to therapy with denosumab or teriparatide. Mutations in the LRP5 gene (lipoprotein receptor 5) are associated with changes in the activity of the WNT signaling pathway, which is important for osteogenesis [191]. Patients with certain LRP5 variants may respond better to drugs that stimulate this pathway (e.g., romosozumab, a sclerostin inhibitor) [192,193]. Polymorphisms in the ESR1 gene (estrogen receptor α) gene affect the response to hormone replacement therapy (HRT) [194] and selective estrogen receptor modulators (SERMs, e.g., raloxifene) [195]; some ESR1 variants were reported to associate with a better response to HRT in postmenopausal women [196,197].

A promising area of research in the field of targeted osteoporosis therapy deals with the development of nucleic acid-based drugs, such as siRNA, antisense oligonucleotides, and aptamers, which possess high specificity and the potential to modulate key regulatory pathways of bone remodeling. siRNAs and antisense oligonucleotides allow for the targeted manipulation of the expression of genes associated with osteoclastic activity, osteoblastic stimulation, and the balance between them, while aptamers can inhibit the functional activity of regulatory proteins, playing the same role as monoclonal antibodies [198].

One of the most actively researched areas is the use of siRNA (small interfering RNA) to suppress the expression of genes responsible for osteoclast activity, such as human RANKL (TNFSF11) [199]. Some examples of siRNAs capable of downregulating OP-associated genes are given in Table 2.

Preclinical studies demonstrated that the administration of specific siRNAs significantly reduces osteoclastic activity and promotes an increase in bone density [199]. In particular, studies in mouse osteoporosis models demonstrated that locally or systemically delivered siRNA can significantly reduce the risk of bone fractures and improve bone microstructure [123].

Speaking of siRNA, we should also mention miRNA as an alternative targeted nucleic acid regulating gene expression at the mRNA level. Studies on osteoporotic mouse models revealed that the AAV9 vectors (recombinant adeno-associated viral vectors) coding miRNAs can suppress the expression of the SHN3 and SOST genes, thus activating the Wnt pathway [134].

Antisense oligonucleotides (ASO) block the transcription or translation of target genes by means of complementary interactions [198]. In contrast to siRNAs, only solitary examples of antisense oligonucleotides targeting DKK-1 in the context of bone diseases have been reported [135,200,201].

Aptamers are short synthetic nucleic acids with high affinity and selectivity for certain target molecules, including soluble regulatory proteins. Having the same mode of action as monoclonal antibodies, aptamers enjoy the advantages of a precisely known nucleotide sequence, reproducible chemical synthesis with negligible batch-to-batch variation, low immunogenicity, and long shelf-life and do not require cold chain transportation [202,203]. In osteoporosis research, aptamers could be used to block factors that regulate bone remodeling, such as WNT pathway proteins or molecules involved in osteoclast regulation [204]. To date, the most promising example of anti-OP aptamers is the aptamer targeting sclerostin [136], which stimulated osteoblast activity, increased BMD, and improved bone microarchitecture and biomechanics, as confirmed by in vivo studies on OP mice models [205,206]. Its use in clinical settings promises to provide more accurate and safer effects due to its high specificity.

An important aspect of the development of nucleic acid-based molecular therapies is the improvement of delivery methods, as nucleic acids require protection from degradation in vivo, optimal pharmacokinetic properties, and addressed delivery to bone tissue. Multiple nanotechnology platforms, such as liposomes, nanoparticles, and modified carriers, provide stability and selectivity of delivery [207]. Namely, nanoparticles coated with hydroxyapatite or osteonectin allow for the targeted delivery of siRNA to bone tissue, minimizing systemic side effects and increasing the treatment efficacy [208].

In addition, ongoing studies also explore the possibility of combining targeted nucleic acids with traditional therapies, such as bisphosphonates or monoclonal antibodies, to enhance their synergistic effect and reduce dose loads. Work is also underway to develop systems that allow for time- and intensity-controlled nucleic acid release for achieving optimal therapeutic results [209].

Despite significant progress, a lot of challenges remain related to the safety, immunogenic reactivity, and stability of nucleic acid drugs. In this regard, research is continuing to improve chemical modification and develop new delivery technologies, which will expand their application in clinical practice and increase their therapeutic potential [113]. 

Small molecules represent an important class of new therapeutic agents for the treatment of osteoporosis, capable of targeted action on key signaling pathways that regulate the balance between bone formation and bone resorption. Unlike biological drugs, small molecules possess advantages such as high bioavailability, the possibility of oral administration, and lower production costs, which makes them particularly attractive for long-term therapy [210].

One of the most promising areas is the development of inhibitors of enzymes involved in bone matrix degradation. Among them, compounds that inhibit cathepsins, a family of proteases responsible for the breakdown of collagen and other bone tissue components, look especially attractive. Preclinical models revealed effective cathepsin inhibitors, which help to preserve the bone structural integrity and increase BMD [211].

Small molecules that affect the WNT/β-catenin and RANK/RANKL signaling pathways are also of considerable interest. Compounds of this type are capable of modulating the activity of the above-mentioned pathways, stimulating osteoblastic function and suppressing osteoclast activity [212]. For example, some small molecules that inhibit WNT pathway proteins have been shown to be effective in increasing bone mass and reducing the risk of fractures in osteoporosis models.

Another important area of research is creating inhibitors of enzymes involved in the metabolism of biomolecules, especially those responsible for the degradation of collagen and other components of the bone matrix. In particular, molecules that block protein kinases involved in the regulation of osteoclast and osteoblast cell activity can reduce bone loss and promote bone tissue regeneration, which is particularly relevant in severe forms of osteoporosis [213].

Despite their obvious advantages, the development of small molecules faces a number of challenges related to ensuring their high selectivity and minimizing side effects. In this regard, research is continuing on optimizing the chemical structure and developing new methods for assessing pharmacokinetics and pharmacodynamics [214]. In the future, these molecules are expected to occupy an important place in the arsenal of anti-OP therapeutic agents.

Nanomaterials can provide the controlled delivery of small molecule drugs to the bone tissue [214]. This approach allows for the precise placing of therapeutic agents to the site of the pathological process, which is particularly important in the treatment of osteoporosis.

Nanoparticles can carry low-molecular-weight compounds, such as inhibitors of enzymes that regulate bone remodeling, or biological agents that stimulate osteoblastic activity [215]. Nanosystems containing inhibitors of collagen degradation enzymes or protein kinases have demonstrated the ability to stabilize bone structure in the long-term at minimal systemic drug concentrations [208]. This effect originates from the high concentration of active substances inside the nanoparticles and their targeted release into bone tissue.

Targeted delivery nanoparticles also make possible the use of low-molecular-weight compounds, which under normal conditions have low bioavailability and high toxicity at systemic administration. The introduction of nanoparticles loaded with collagen-degrading enzyme inhibitors provided their stability in the bloodstream and effective penetration into bone cells [216].

Nanoparticle-assisted delivery also allows for controlled drug release. Varying the size, shape, and surface characteristics of nanoparticles makes it possible to regulate the rate of release, providing a prolonged effect and reducing the frequency of administration [217]. Namely, polymer-coated nanoparticles serve as a basis for multiphase systems that release active components over several weeks, which could significantly improve patient comfort and promote compliance with the treatment regimen.

We should also mention actively developing multifunctional nanoparticles that combine several functions: targeted delivery, controlled release, and visualization of the therapy process. Such systems can bear labels of different types, e.g., fluorescent ones, giving the possibility of the real-time monitoring of distribution and delivery efficiency [218].

Despite significant progress, there are still a number of technical and regulatory challenges related to production scaling, nanoparticle stability, and safety. In particular, further research is needed to assess the long-term biocompatibility and potential toxicity of nanomaterials [219]. Another important aspect is the lack of standard methods for assessing the quality of nanoparticles and their pharmacokinetics, which will ensure their safety and efficacy in clinical practice.

## 6. Conclusions

Osteoporosis remains an acute problem of health and a challenge for researchers developing novel therapy strategies. We reviewed here clinical aspects of OP, the main pathways of its pathogenesis, and the medicines routinely used to prevent bone fragility, either by reducing bone resorption or through osteoanabolic effect. Despite numerous advantages, existing drugs also possess some drawbacks that could become critical in certain cases. Moreover, in patients at very high fracture risk, it is crucial not only to slow down bone mass and density but also to increase them quickly and safely. Only precise, targeted action on OP pathogenesis pathways provides reliable protection from fractures while avoiding undesirable events. Two targeted drugs have been approved for clinical use for OP, denosumab (RANKL inhibitor) and romosozumab (sclerostin inhibitor). Despite their great importance, they are also not free of adverse effects, such as the rebound effect for denosumab or cardiovascular risks for romosozumab. Thus, the development of novel, highly efficient, and safe pharmacological approaches for OP treatment still represent an important scientific and practical task. Researchers and clinicians have investigated novel points of action (inhibition of cathepsin K, TGF-β, and DKK-1) and examined new ways to regulate already known targets. In particular, oligonucleotide aptamers promise to enlarge a toolkit of targeted biomolecules, in addition to monoclonal antibodies. Aptamers provide unique benefits such as reproducible chemical synthesis, do not require cold chain transport, and could potentially suit continuous drug delivery devices, facilitating risk and efficacy management. Nanomaterials show significant potential for targeted drug delivery, and controlled release also looks very promising. Furthermore, the use of pharmacogenomic methods makes it possible to develop personalized strategies of OP treatment. Given the high and permanently growing incidence of OP in the population and its negative consequences for both individuals and society as a whole, the search for new therapeutic strategies is crucial for reducing mortality, preventing disability, and prolonging active life.

## Figures and Tables

**Table 1:** Different classes of anti-osteoporotic molecules in clinical practice and under development.

Class of Anti-Osteoporotic Drug (International Nonproprietary Names)	Mechanism	Efficacy	Side Effects	Clinical Status
Anti-resorptive drugs				
Bisphosphonates (zoledronate, alendronate, ibandronate, risedronate)	Inhibit hydroxyapatite crystals destruction. Suppress osteoclasts (through cell death due to toxic ATP analogues (nitrogen-free bisphosphonates)/promote osteoclast apoptosis by inhibiting FPPS (nitrogen-containing bisphosphonates) [101].	Increase BMD values and reducing the risk of osteoporotic fractures. First-line drugs.	Oral administration: heartburn, indigestion, esophageal erosion, and ulcers. Parenteral administration: flu-like symptoms (fever, muscle pain, and arthralgia), hypocalcemia. Rare: excessive bone fragility, jaw osteonecrosis [11,102] Potential teratogenicity/placental permeability.	Phase 4 (post-market)
Strontium ranelate	Increases osteoblasts proliferation and differentiation. Incorporates into bone cells, increasing their density. Suppresses the formation and differentiation of osteoclasts and promotes their apoptosis [103].	Significant bone mass increasing. Useful as a component of modified biomaterials [103]. Second-line drug.	Severe skin reactions (toxic epidermal necrolysis), cardiovascular disease, thromboembolic complications, hypocalcemia [103,104].	Phase 4 (post-market)
Calcitonin	Causes osteoclasts’ outflow from areas of active bone resorption. Inhibits the differentiation and proliferation of osteoclasts [13,105].	The resorption of bone matrix is diminished. Reduces solely the risk of vertebral fractures and only when administered nasally Has analgesic effect [13,106,107]. Second-line drug.	Nasal discomfort, abnormal product odor, nausea, loss of appetite, diarrhea, vomiting, abdominal pain, hot flushes, hypocalcemia, allergic reactions [108,109].	Phase 4 (post-market)
Estrogen- Progestin Therapy	Compensates for the hormone deficiency and reduces bone loss by suppressing osteoclast activity [110].	Reduces estrogen deficiency-mediated increased bone turnover and prevent further bone loss. Significantly reduces the risks of hip and vertebral fractures [111,112]. Second-line drug.	High risk of cardiovascular disease, stroke, venous thromboembolism, invasive breast cancer [113].	Phase 4 (post-market)
SERMs (raloxifene, bazedoxifene)	Induces osteoclast apoptosis. Act as estrogenic agonists in bone [112].	Inhibits accelerated bone resorption in both the short- and long-term, increases BMD, and bone strength. Prevents bone loss and reduces the risk of spinal fractures, but not fractures in other locations [114]. Second-line drug.	Risk of deep vein thrombosis, pulmonary embolism, vaginal bleeding, stroke, cardiovascular disease, hot flashes, leg cramps [11,115].	Phase 4 (post-market)
Anti- RANKL agent (denosumab)	Blocks RANKL, an essential factor regulating osteoclast activity [116].	Reduction in the risk of hip and vertebral fractures with long-term use [117]. First-line drugs.	“Rebound effect” (increase in bone turnover overriding pre-treatment status, a rapid bone loss in the majority and multiple vertebral fracture), hypocalcemia, cellulitis, musculoskeletal pain, jaw osteonecrosis [11,102,118,119].	Phase 4 (post-market)
Cathepsin K inhibitors (odanacatib, balicatib)	Limits osteoclast activity without suppression of osteoblast function [120].	Reduces the risk of hip and spine fractures due to increasing in BMD and improving bone strength at the spine and hip [121].	High risk of stroke, probable pycnodystosis [121,122].	Phase 3
RANKL- suppressing siRNA [123]	Reduction in osteoclast formation.	Reduction in bone loss, BMD increase.	No data available.	Pre-clinical research
BTK inhibitor [124]	Inhibits the M-CSF and RANKL-driven osteoclast differentiation.	Suppresses bone loss in mice.	No data available.	Pre-clinical research
p38 MAP kinase inhibitor [125]	Represses RANKL-induced osteoclast differentiation.	Prevents bone loss in ovariectomized mouse mode by inhibiting both bone resorption and bone formation in vivo.	No data available.	Pre-clinical research
Anabolic drugs				
PTH analogues (teriparatide, abaloparatide)	Increases osteoblastic activity through binding with the parathyroid hormone receptor 1 (PTHR1) [126].	Stimulates bone formation. Prevents non-vertebral fractures and improving spine BMD [12,127].	Probable osteosarcoma risk, cephalgia, dizziness, limb cramps, nausea, hypercalcemia [10,11,12,128].	Phase 4 (post-market)
Anti-sclerostin agent (romosozumab)	Inhibits the binding of sclerostin to the LRP 5/6-frizzled receptor complex, thereby activating the Wnt signaling pathway [118].	Rapid increases in bone mineral density. Significant reductions in the risk of fractures Loss of anabolic effect after stopping treatment [118].	Arthralgia, nasopharyngitis, injection-site reactions, headache, cataracts, risk of cardiac events, hypocalcemia, osteonecrosis of the jaw, atypical femur fracture, serious infections for elderly patients [118].	Phase 4 (post-market)
Anti-sclerostin agent (blosozumab)	Binds directly to sclerostin, which is an inhibitor of the Wnt signaling pathway [129].	Improves lumbar bone mass in postmenopausal women [130].	Arthralgia, back pain, fatigue, headache, injection site reactions, nasal congestion, nausea, upper respiratory tract infection, and vomiting [118,129,130].	Phase 2
Bispecific antibodies blocking RANKL and sclerostin [131]	Allows for both the suppression of osteoclastic activity and stimulation of osteoblastic function.	No data available.	No data available.	Pre-clinical research
siRNA targeting WNT antagonists (DKK-1, SOST) [132,133]	Activation of the Wnt pathway (increase in β-catenin level). Increased mineralization of osteoblasts.	Increase BMD, mineralization, trabecular bone, decrease osteolysis in mice. Provide reduction in fracture frequency.	No data available.	Pre-clinical research
miRNA [134]	Suppress the expression of the SHN3 and SOST genes, thus activate the Wnt pathway.	Increases in trabecular bone mass in mice.	No data available.	Pre-clinical research
Antisense oligonucleotides targeting DKK-1 [135]	Activation of the Wnt pathway. Stimulate osteoblast activity. Reduce ovariectomy promotion of ex vivo osteoclast differentiation of primary M-CSF-dependent bone marrow macrophages. Increase osteoblast number.	Reduce bone loss and improves the biomechanical properties of bone in mice.	No data available.	Pre-clinical research
Aptamers targeting sclerostin [136]	Attenuate inhibitory effect of SOST on bone formation.	Promote bone formation in mice and ovariectomy-induced osteoporotic rats.	No data available.	Pre-clinical research

**Table 2:** Most representative examples of siRNA downregulating the expression of genes responsible for osteoclast activity.

Model	Target	siRNA	Key Findings and Evaluation Methods	References
Human (TNFSF11)	RANKL	siRANKL-1, Sense: 5′-GCAUGAAGACUCCAGAACAdTdT-3′ siRANKL-2, Sense: 5′-CCUGGAAACUGCUGAAAUAdTdT-3′	Molecular level: suppression of RANKL mRNA by 60–80% (qPCR); reduction of RANKL protein level (Western blot/ELISA/IHC). Functional/macro level:—Reduction in osteoclast formation by 50–70%; reduction in bone loss, increase in BMD by 20–30%; reduction in the number of TRAP+ osteoclasts.	[123]
Human (NM_012242)	DKK1	siDKK1-1: Sense: 5′-GCAUGUACUGUGGAUCAUAdTdT-3′ siDKK1-2: Sense: 5′-CCACCAAGUGUACAUCUAUdTdT-3′	Molecular level: suppression of DKK1 mRNA and protein levels by 60–80%; activation of the Wnt pathway (increase in β-catenin level). Functional/macro level:—Increased mineralization of osteoblasts; increase in bone mass by 25–30% (micro-CT); reduction in resorption markers (CTX-1).	[132]
Human (NM_025237)	SOST	siSOST-1: Sense: 5′-GCAUGAAGCUCCUGAAACAdTdT-3′ siSOST-2: Sense: 5′-CCUGGAACUGCCAGAAGAUdTdT-3′	Molecular level:—Suppression of SOST mRNA by 70–90%.—Increased Wnt/β-catenin activity. Functional/macro level:—30% increase in BMD.—Increased mineralization, decreased osteolysis.—Increase in trabecular bone, reduction in fracture frequency.	[133]

## Data Availability

Data sharing is not applicable to this article.

## Abbreviations

The following abbreviations are used in this manuscript:

ABL	Abaloparatide
ALP	Alkaline phosphatase
ASOs	Antisense oligonucleotides
ATP	Adenosintriphosphate
BMD	Bone mineral density
BMSC	Bone marrow-derived MSC
BMP	Bone morphogenetic protein
BMPR	Receptor for Bone morphogenetic protein
BTK	Bruton’s tyrosine kinase
CHO	Chinese Hamster Ovary
CTX-1	Carboxy-terminal crosslinked telopeptide of type 1 collagen
DKK	Dickkopf
DNA	Deoxyribonucleic acid
DXA	Dual-energy X-ray absorptiometry
EPT	Estrogen-Progestin Therapy
ER	Estrogen receptor
EVs	Extracellular vesicles
FDA	Food and Drug Administration
FPPS	Farnesylpyrophosphate synthase
FRAX	Fracture Risk Assessment Tool
FZD	Frizzled
HRT	Hormone replacement therapy
IL	Interleukin
lncRNAs	Long non-coding RNAs
LRP	Low-density lipoprotein receptor-related protein
MMP	Matrix metalloproteinase
MCP-1	Monocyte chemotactic protein 1
MCSF-1	Macrophage colony stimulating factor
MCSFR	Receptor for Macrophage colony stimulating factor
MPA	Medroxyprogesterone acetate
MSC	Mesenchymal stromal cell
oCEE	Oral conjugated equine estrogen
OP	Osteoporosis
OPG	Osteoprotegerin
PINP	Procollagen type I N-terminal propeptide
PPARγ	Peroxisome proliferator-activated receptor gamma
PTH	Parathyroid hormone
RANK	Receptor activator of nuclear factor-κB
RANKL	Receptor activator of NF-κB ligand
ROS	Reactive oxygen species
SASP	Senescence-associated secretory phenomenon
SERMs	Selective estrogen receptor modulators
siRNA	Small interfering RNA
SOST	Sclerostin
TBS	Trabecular Bone Score
TGF-b	Transforming growth factor beta
TNF	Tumor necrosis factor
WHO	World Health Organization
